# Lead toxicity among traffic wardens: a high risk group exposed to atmospheric lead, is it still a cause for concern?

**DOI:** 10.1186/s12995-015-0046-9

**Published:** 2015-02-08

**Authors:** Benedict Samuel Sebastiampillai, Mitrakrishnan Rayno Navinan, Sahan Indeewara Talpe Guruge, Dilushi Rowena Wijayaratne, Buddini Samanthi Dissanayake, Dissanayake Mudiyanselage Sushrutha Vajira Dissanayake, Manisha Samithri Perera, Sulakshi Manurika Thelikorala, Hasith Dhanajaya Wijayasurendra, Don Lasitha Naveen Wickramaratne, Carukshi Arambepola, Ravindra Fernando

**Affiliations:** Faculty of Medicine, University of Colombo, Colombo, Sri Lanka; Department of Community Medicine, Faculty of Medicine, University of Colombo, Colombo, Sri Lanka; Department of Forensic Medicine and Toxicology, Faculty of Medicine, University of Colombo, Colombo, Sri Lanka

**Keywords:** Lead toxicity, Sri Lanka, Unleading, Traffic police

## Abstract

**Background:**

Traffic policemen are identified to be at a higher risk of exposure to air pollution and its contaminants such as lead. A study done prior to the introduction of unleaded petroleum in Sri Lanka revealed a mean blood lead level of 53.07 μg/dL, which was well above the Center for Disease Control defined acceptable safe levels. This study aimed to determine whether unleading of fuel has made an impact on the blood lead levels of traffic police working in an urban area with high traffic density.

**Method:**

A cross-sectional survey of 168 traffic police personnel working within Colombo city limits of Sri Lanka, a high traffic density area, was conducted. Blood lead levels of participants were measured using nitric acid, perchloric acid ashing method and atomic absorption spectrophotometry. An interviewer administered questionnaire was used for a targeted history and examination.

**Results and discussion:**

Mean age of the sample population was 37 years. Thirty eight percent had detectable levels of lead in blood and 24.4% of the study sample had blood lead levels above Centre for disease control defined safe limits. Sample mean was 4.82 μg/dL (95% CI 3.58-6.04), and this is a 91% overall reduction when compared to data prior to unleading. Neither symptoms nor signs of classic lead toxicity showed significant correlation with toxic lead levels.

**Conclusion:**

Lead poisoning though still present in the high risk traffic warden population shows a considerable reduction following unleading. The need to have a low threshold to suspect lead poisoning is highlighted by the non-specific nature of the symptoms and signs of lead poisoning and its lack of association even in those found to have elevated lead levels. Further studies are required to elucidate a cause for the prevalence of lead poisoning despite cessation of using lead as an additive in petroleum.

## Background

Traffic policemen have been previously identified as at a higher risk of environmental air pollution including lead toxicity due to exposure to atmospheric lead contaminants [1] to which combustion of leaded petroleum was a significant contributor prior to it being unleaded [2].Leaded petroleum was used in Sri Lanka until 2002. In 1996 prior to unleading of fuel, a study by Arewgoda revealed that Sri Lankan policemen had markedly elevated blood lead levels, with an overall mean of 53.07 μg/dL [3]. This was higher than the Centre for Disease Control (CDC) recommended maximum safe blood lead level of 10.00 μg/dL. Amarasinghe in 2002 compared blood lead levels among traffic police and non-traffic policemen and women. Though his analysis revealed both groups to have elevated blood lead levels, there was no statistically significant difference between the two groups [4]. The only study done in Sri Lanka to determine the impact of unleading of petroleum was conducted in children and it showed low blood lead levels following unleading, below the accepted CDC safe limit [5]. This study is the first one conducted in Sri Lanka to determine if unleading of petroleum had made an impact on the blood lead levels among a population previously at high risk of exposure.

## Method

Traffic wardens working within the urban city limits in areas identified as having high traffic density (based on data obtained from the Transportation Engineering Division, University of Moratuwa, Sri Lanka) were considered eligible for this study. A minimum period of active service in the field for at least 6 months or greater was used as an inclusion criterion. Individuals who were already diagnosed with lead toxicity or those who recently transferred in from areas outside of the defined study area and those who refused consent were excluded. Amongst the traffic wardens who were assessed for suitability only 168 were found eligible and were included in this cross-sectional analysis. Trained phlebotomists collected blood under aseptic conditions. The containers used to collect blood were verified as lead free by the same laboratory that analysed the samples to avoid contamination. 10 ml of blood was collected and stored from those who consented and were labelled and transported immediately in ice as per laboratory norms. Blood lead levels of participants were measured using nitric acid, perchloric acid ashing method and graphite furnace atomic absorption spectrophotometry (Model GBC 932, GBC Scientific, Dandenong, Victoria, Australia). The atomic absorption spectrophotometer was calibrated using commercially available standard lead solution having a concentration of 1000 ppm and the method detection limit of 0.01 μg/dL was established by repeated double dilution of the standard lead solution.

An interviewer administered questionnaire was utilized to obtain data from participants with regards to their socio-demographic information, use of personal protective equipment, exposure to other potential sources of lead including environmental exposure, use of Ayurvedic (indigenous) medication and the presence of any symptoms of lead toxicity (Table 1). The above factors were defined as binary or categorical variables as appropriate. A targeted clinical examination was conducted for signs of chronic lead toxicity by the investigators. The signs considered were pallor, blue line on gums and features of peripheral motor neuropathy (i.e. wrist drop and foot drop).

**Table 1:** **Distribution of common symptoms suggestive of lead toxicity and its association between assessed blood lead levels**

**Symptoms**	**Blood lead level <10 ug/dl**		**Blood lead level >10 ug/dl**		**χ** ^**2**^	**p-value**
	**Present**	**Absent**	**Present**	**Absent**		
	**n (%)**	**n (%)**	**n (%)**	**n (%)**		
Frequent headache	31 (18.4)	96 (58.3)	7 (4.1)	34 (20.2)	0.953	0.329
Fatigue	29 (17.2)	98 (58.3)	7(4.1)	34 (20.2)	0.611	0.434
Tremors	11 (6.5)	116 (69.8)	2 (1.2)	39 (23.2)	0.621	0.431
Irritability	18 (10.7)	109 (64.8)	6 (3.5)	35 (20.8)	0.005	0.942
Nausea	3 (1.7)	124 (73.8)	1 (0.5)	40 (23.8)	0.001	0.978
Constipation	10 (5.9)	117 (69.6)	2 (1.2)	39 (24.2)	0.419	0.517
Insomnia	7 (4.1)	120 (71.4)	3 (1.7)	38 (22.6)	0.180	0.671

(Total sample size 168).

Data was evaluated using univariate and bivariate analysis using statistical software SPSS version 17.0 (SPSS Inc., Chicago, IL, USA). Pearson chi square was used to assess relationships between categorical variables. Statistical tests were considered to be significant at an alpha level of 0.05 on a two-tailed test.

## Results

Hundred and sixty eight (168) traffic police officers consented to participate in the study. The mean age was 37 years. Majority (61.9%, n = 104) did not have detectable levels of lead in blood (<0.01 μg/dL) and of the 38.1% (n = 64) who had detectable levels of lead in blood, 35.9% (n = 23) had levels below the accepted CDC safe limit (<10.00 μg/dL). Of the 41 whose blood lead level exceeded CDC defined safety limit, the highest observed value was 54.50 μg/dL. This distribution of blood lead levels resulted in a sample mean of 4.82 μg/dL (95% CI 3.58-6.04). Exposure to lead through to lead based industries (p = 0.424), smoking cigarettes (p = 0.071), drinking pipe borne water carried through old water systems before the advent of lead free pipes (p = 0.362) did not show any correlation with blood lead levels. Nor did the use of personal protective equipment (p = 0.804) have a significant impact blood lead levels. The presence of pallor (n = 10), peripheral neuropathy (n = 3) and blue lines in gums (n = 2) in the study population did not correlate significantly with elevated blood lead levels in the population (p values = 0.084, 0.716, 0.419 respectively for each sign). The presence of symptoms from the catalogue of classic symptoms of lead toxicity did not correlate in a statistically significant manner to elevated blood lead levels (Table 1). Individuals who were found to have elevated toxic lead levels were referred for further treatment to a tertiary care hospital.

## Discussion

As Sri Lanka has taken steps to discontinue the addition of lead to petroleum since 2002 we hypothesized that vehicular emissions as a source of lead should be absent in the current setting. Thus we postulated that the removal of a principle source of lead poisoning would lead to an impact on blood lead levels in traffic police, a population previously at high risk of exposure to atmospheric lead pollutants.

Our study’s sample mean of 4.82 μg/dL was well below clinically toxic levels in comparison to a much higher recorded mean value of 53.07 μg/dL reported by Arewgoda in 1996 prior to the introduction of unleaded petroleum [3]. While direct comparisons between previous studies and the current study are not possible due to variations in sample selection between the studies, a general inference may be made as to effectiveness of removal of lead additives from petroleum leading to an overall reduction of observed lead toxicity by as much as 91%, which is to be expected. However it should still be noted 24.4% (n = 41) of the study sample had blood lead levels above the safe limit, an alarming finding in a population that was previously at risk. Though we considered documented external factors (smoking, water contamination, industry exposure) which are known to be sources of lead toxicity [6-8] other than atmospheric pollution, lead levels in our study failed to demonstrate a correlation with these factors.

This study was limited as samples were obtained from a relatively low number of volunteers from a single metropolitan area with high traffic density, which limits its ability to provide an overall picture of the pattern and prevalence of this problem at the national level. However it should be noted that the metropolitan area the samples were obtained from in this study has the highest population density in the island with an attendant high traffic density. In consideration of the high risk populace analysed in this study, it shows that lead poisoning is still prevalent despite the cessation of a significant source of environmental lead pollution, similar to what was seen by Vishwanath et al. [8]. This implies that there may be other, as yet unascertained, sources of lead pollution present in environment which needs identification.

With the lack of appreciation of the prevalence of lead toxicity and this being further compounded by the ambiguity of the classic symptoms, individuals with lead poisoning may go unnoticed. This along with the long term detrimental health effects brought about by lead poisoning, may unnecessarily burden the health sector. Clinical suspicion should remain high when a myriad of non-specific complaints are present, especially in a classic high risk population. Lead poisoning is still prevalent and is a problem that has not been fully addressed.

## Conclusion

In a high risk group such as traffic police wardens, lead toxicity though still present shows a marked decline in mean blood lead levels by as much as 91%, compared to the levels found prior to removal of lead from petroleum. The need to have a high index of suspicion in detecting lead poisoning in an at-risk population is highlighted by its non-specific symptoms and signs and their lack of correlation to blood lead levels. Further studies are required to elucidate a cause as to why lead poisoning is prevalent with blood lead levels seen well above safe limits despite steps taken to counter this through unleading of petroleum. Lead poisoning is problem that remains to be fully addressed.

## References

[1] Mortada WI, Sobh MA, El-Defrawy MM, Farahat SE (2001). Study of lead exposure from automobile exhaust as a risk for nephrotoxicity among traffic policemen. Am J Nephrol.

[2] Eisenreich SJ, Metzer NA, Urban NR, Robbins JA (1986). Response of atmospheric lead to decreased use of lead in gasoline. Environ Sci Technol.

[3] Arewgoda CM, Perera MS (1996). Blood lead levels in a population exposed to vehicle emissions. Journal of the National Science Council of Sri Lanka.

[4] Amarasinghe, JNP. Blood lead levels of traffic policeman in the city of Colombo. PGIM Theses and Dissertations 2002; Available from: http://pgim.srilankahealthbiblio.org/handle/123456789/19978

[5] Senanayake MP, Rodrigo MD, Malkanthi R (2004). Blood lead levels of children before and after introduction of unleaded petrol. Ceylon Med J.

[6] al-Saleh IA (1995). Lead exposure in Saudi Arabia and its relationship to smoking. Biometals.

[7] Shaik AP, Sultana SA, Alsaeed AH (2014). Lead Exposure: A Summary of Global Studies and the Need for New Studies from Saudi Arabia. Dis Markers.

[8] Vishwanath P (2012). Environmental lead levels in a coastal city of India: the lead burden continues. Indian J Med Sci.

